# Electropolymerized polypyrrole-MOF composite as a coating material for SPME fiber for extraction VOCs liberated by bacteria

**DOI:** 10.1038/s41598-023-36081-9

**Published:** 2023-06-01

**Authors:** Radik Mametov, Gulyaim Sagandykova, Maciej Monedeiro-Milanowski, Dorota Gabryś, Paweł Pomastowski

**Affiliations:** 1 Centre for Modern Interdisciplinary Technologies, Nicolaus Copernicus University in Toruń, Wileńska 4, 87-100 Toruń, Poland; 2 Radiotherapy Department, Maria Sklodowska-Curie National Research and Institute of Oncology, Gliwice, Poland

**Keywords:** Analytical chemistry, Bioanalytical chemistry, Materials science, Biomarkers, Bacteria

## Abstract

The synthesis of efficient and low-cost coatings for solid-phase microextraction attracted much attention. Conductive polymers are excellent candidates for this purpose due to the possibility of electropolymerization, which results in the reproducible synthesis of films. A plethora of studies reported in the literature concluded that modification of conductive polymers with innovative materials could lead to an increase in sensitivity toward specific analytes. In this work, the metal–organic framework-polypyrrole composite was electrodeposited in one step directly onto a stainless-steel substrate. The effect of synthesis parameters on extraction efficiency was investigated. The obtained PPy@ZIF-8 coating was subjected to physical–chemical characterization using electron microscopy and Fourier-transform IR spectroscopy. The main finding of the study was that the values of the limit of detection and intra- and inter-day reproducibility for analytes with different chemical structures were found to be lower as compared to pure polypyrrole coating. Furthermore, the obtained polypyrrole-MOF coating was applied for the collection of profiles of volatile organic compounds liberated by bacteria. Hence, the polypyrrole@ZIF-8 coating synthesized using a low-cost and facile approach presented in this study can be useful for the profiling of VOCs liberated by bacteria.

## Introduction

Solid-phase microextraction (SPME) techniques are facile and solvent-free in comparison to exhaustive traditional sample preparation technologies^1,2^. Since its first introduction in 1990, developing analytical methods based on SPME for various applications is a constantly evolving research area. Diverse SPME tools were successfully introduced, such as in-tube SPME, needle trap, and single-drop microextraction^3^.

SPME is useful in many fields, such as environmental research, food chemistry, and pharmaceutical and biomedical analysis^4^. One of the relatively recent applications of SPME concerns the analysis of volatile organic compounds emitted by bacteria.

Traditional methods for the identification of pathogens, such as culturing and biochemical tests, suffer from numerous limitations. The major disadvantage is that the procedure is laborious and time-consuming^5^. Nucleic-based and matrix-assisted laser desorption/ionization techniques tackle the limitations of traditional methods offering rapid and accurate identification^6^. However, there is a constant demand for the development of analytical techniques for bacterial identification since metabolic alterations of bacterial species complicate identification.

To the best of our knowledge, the identification of volatile organic compounds (VOCs) is an opportunistic approach to the detection of pathogenic bacteria^7^ and a way to understand different metabolomic processes^8^. The advantage of VOCs’ profiling is uncomplicated sample preparation. Extraction methods such as headspace solid-phase microextraction (HS-SPME), thermal desorption, purge-and-trap, and needle trap are used for profiling bacterial VOCs^9^. Headspace solid-phase microextraction (HS-SPME) is a popular extraction method for profiling bacterial VOCs, combining extraction and pre-concentration in one step.

However, the application of SPME in biomedical research requires low values of a limit of detection. This leads to the development of SPME fibers based on various materials. A few types of materials were presented, such as ionic liquids, metal–organic frameworks (MOF), conductive polymers, nanomaterials, and their composites^10^. In addition, commercially available fibers have some disadvantages, such as lack of thermal stability, high price, and fragility. For this reason, developing new types of SPME fibers on the stainless steel wire with new sorbent types is actual^11^.

Hence, the study aims to report a facile, low-cost method for the electropolymerization of polypyrrole-MOF composite as a coating material for SPME fiber. The new-coated SPME fibers will be used to extract VOCs emitted by bacteria.

Conductive polymers are favourable for application as a coating material for SPME. The materials’ multifaceted properties include polar functional groups, electroactivity, π–π interactions, and hydrophobicity^12^. The most widely used representatives are polypyrrole, polyaniline, polythiophene, and composites based on them^11^. For the study, polypyrrole was chosen as a base for the new coatings, since our research team has already reported about electropolymerized PPY-coating for SPME fibers^13^.

The metal–organic framework Basolite Z1200 (ZIF-8) was used as a modification agent to improve the extraction efficiency of PPy-based fibers.

MOFs are hybrid inorganic–organic materials constructed from organic ligands and metal ions. Various metal ions and organic linkers make it possible to synthesize massive types of new MOFs with different structures, surface area, porosity, etc. Nowadays, MOFs are used for various applications in analytical chemistry^14^. In particular, there is a considerable amount of studies on applications of MOFs as sorbent materials for the different extraction and separation techniques. Examples are applications in the solid-phase extraction as a sorbent, the filling/support materials for the chromatographic columns, the microextraction techniques, etc.^15^.

Zeolite imidazole’s frameworks (ZIFs) are a subclass of MOF materials. ZIFs are formed by the transition of metal ions (Zn) and imidazolates linkers^16^. In particular, ZIF-8^16^ has high thermal and mechanical stability and a surface area > 2000 cm^2^ g^−1^. In addition, ZIF-8 is a water-stable MOF, which prevents loss of sensitivity since water occupies the sorption sites interfering with analytes. Furthermore, this allows for applications of the coating not only for headspace SPME, but also for direct immersion SPME from solution, thus extending the application field.

Notably, the adhesion of MOFs towards metallic substrate is a limitation for its use as the coating for SPME fibers. Few studies addressed the problem by synthesis of polydimethylsiloxane (PDMS)^16^. This means that hybrid composites can tackle the limitations of a single material.

Rare efforts have been made to synthesize SPME fibers based on conductive polymers with metal–organic frameworks. Bagheri et al.^17^ reported headspace-SPME fibers based on polyaniline-MOF nanocomposite synthesized with electropolymerization in one step.

## Experimental

### Reagents and materials

Basolite Z1200 (2-Methylimidazole zinc salt, ZIF-8), Pyrrole (98%), Brain Heart Infusion Agar (BHIA), sodium dodecylbenzene sulfonate, sodium perchlorate, acetonitrile (≥ 99.9%), methanol (≥ 99.9%), acetone (≥ 99.5%), hydrofluoric acid (48%), benzene, toluene, p-xylene, ethylbenzene, dodecane, phenol and 1-bromo-4-fluorobenzene (BFB) as analytical standards were all purchased from Sigma-Aldrich. Water was obtained using the Milli-Q RG apparatus by Millipore (Millipore Intertech, Bedford, MA, USA). Stainless steel wire (d = 0.1 mm) was purchased from a commercial source (BS Niżpol, Przemyśl, Poland).

Gas chromatographic analyses for standard solutions were performed with a gas chromatograph GC 7820A coupled to a flame ionization detector (Agilent, Santa Clara, CA, USA). The GC was equipped with an HP-5 analytical column (30 m × 0.32 mm i.d. and film thickness 0.25 μm). Helium (99.99%) and air were used as carrier and make-up gases, respectively. Injections were performed in the splitless mode. GC injector port operated at 220 °C. The initial oven temperature was 30 °C (held for 3 min), ramped at a rate of 4 °C min^−1^ to 50 °C (held for 1 min), then increased to 70, 100 (held for 1 min), and 200 °C at the rates of 5 °C min^−1^, 7 °C min^−1^ and 40 °C min^−1^, respectively. The final oven temperature was kept for 3 min. Make-up gas, hydrogen, and synthetic air flow were maintained at 30 mL/min, 30 mL/min, and 300 mL/min, respectively. The carrier gas flow rate was 2.4 mL/min. The detector operated at 300 °C.

Gas chromatographic analyses for the VOCs liberated by bacteria were carried out with a gas chromatograph GC 7820A coupled with a mass spectrometer Agilent 5977B MSD (Agilent Technologies, Waldbronn, Germany). The GC was equipped with a DB-5 MS capillary column (30 m × 0.25 mm × 0.25 μm). Helium was used as the carrier gas in constant flow mode at 1 ml/min. Injections were performed in spitless mode. GC injector port operated at 220 °C. The initial oven temperature was 40 °C (held for 4 min), ramped at a rate of 7 °C min^−1^ to 150 °C (held for 2 min), then increased to 250 °C at a rate 10 °C min^−1^. The final oven temperature was kept for 5 min.

The mass spectrometer was operating in the EI (70 eV) mode. The ion source temperature was set to 230 °C, and the transfer line was set to 250 °C; acquisition frequency was set at 2.9 scans/s, and the mass range was 35–550 a.m.u. Compounds were identified by comparing their mass spectra with those contained in the NIST mass spectral library version 2017; each peak was searched manually (including baseline subtraction and averaging over a peak).

### Electrochemical synthesis of the PPY@ZIF-8 coating

Stainless-steel wire was etched with hydrofluoric acid for 30 min at 40 °C to increase the surface area, consequently improving the adhesion between substrate and coatings^15^. Then it was washed with distilled water and dried for 30 min at room temperature. In the next step, the wire was immersed in the beaker with methanol and placed in the ultrasonic bath for 10 min. The procedure was repeated with acetone, followed by drying in nitrogen flow. Polypyrrole-MOF composite was directly electrodeposited on the prepared stainless steel substrate. An electrochemical cell consists of a working electrode (etched SS wire) and a counter electrode SS wire twisted into a circle. To the best of our knowledge, MOFs are insoluble in our working media^17^. A specific amount of the MOF was dispersed in the 0.1 M pyrrole solution and stirred in the ultrasonic bath for 10 min to obtain the homogenous suspension. In the next step, the suspension was added to the electrolyte mixture consists acetonitrile/water (50:50), NaClO_4_ (7 mM), C_18_H_29_NaO_3_S (7 mM), 0.1 M pyrrole monomer, and 0.006 g of MOF. Then SS wire (13 mm) was inserted into the reactive solution as the working electrode. A constant potential of 5 V was applied for different times from 15 to 45 min. After deposition, the fiber was sequentially washed with methanol and distilled water to remove excess of the reaction mixture and dried at room temperature for 2 h. Then the coated polypyrrole-MOF wire was connected to a lab-made SPME holder. Further, the fiber was conditioned in the GC injector port for 2 h at 200 °C using a 25 mL/min helium flow and then for 2 h more at 220 °C using a 35 mL/min helium flow. After this procedure, a clean blank was obtained, and analyses of blank samples were repeated after every three runs of standard samples to ensure the absence of contaminants.

### Physico-chemical characterization of synthesized coating material

#### Scanning electron microscopy

Surface morphology characterization of the polypyrrole-MOF coating was carried out by scanning electron microscope (SEM, LEO 1430 VP, Leo Electron Microscopy Ltd., Cambridge, United Kingdom).

#### Fourier-transform infrared spectroscopy

FTIR spectra of synthesized coating material were obtained by using an FTIR spectrophotometer with attenuated total reflectance (FTIR-ATR) using a Nicolet FT-IR apparatus (Thermo Fisher Scientific, Avatar 360 Omnic Sampler) from 4000 to 500 cm^−1^.

### Utilization of obtained fiber for extraction of VOCs from standard solution

A stock solution containing benzene, toluene, ethylbenzene, *p*-xylene, phenol, dodecane, and BFB was prepared in methanol with a concentration of 1 mg mL^−1^ for each compound. 50 µL of stock solution was spiked in 2950 µL of distilled water to rich approximate concertation of each analyte 16.66 µg mL^−1^. All extraction procedures were performed in 20-mL headspace vials with PTFE septa. Initially, a magnetic stirring bar (10 mm) and 1 g of sodium chloride were added to each vial and closed to prevent sample losses. The vials were then placed on the magnetic stirrer with the heating plate. Extraction parameters included a temperature of 30 °C, a pre-incubation time of 17 min, an extraction time of 49 min, stirring at 750 rpm, and the addition of 1 g of sodium chloride. Desorption was carried out at a temperature 220 °C for 5 min. The parameters of extraction for the selected group of VOCs were optimized in our previous work by using Box–Behnken design response surface method^13^. This method allowed us to compare the efficiency of the new composite coating with different synthesis parameters, as well as to understand the affinity of the coatings for different groups of analytes and the potential relationships between them.

#### Determination of limit of detection, intra-, and inter-assay reproducibility

The limit of detection (LOD) values were determined by analysis of standard solutions with decreasing concentrations of analytes. The solutions were prepared by successive dilutions of a stock solution at a concentration of 17 μg mL^−1^ for each analyte in triplicate. Detection limits were defined as the lowest concentration that provided a signal-to-noise ratio of at least 3 and was significantly different from the blank. Inter-assay reproducibility was determined by analysis of the stock solution for three consecutive days. Intra-assay reproducibility was determined by analysis of the stock solution in triplicate.

### Application of the obtained fiber for extraction of VOCs emitted by bacteria

Three species of bacteria (*Hafnia alvei, Proteus mirabilis, Enterococcus faecalis and Morganella morgani*) were isolated from feces samples of patients with diagnosed colorectal cancer. Biological material was plated on Petri dishes with Brain Heart Infusion Agar (BHIA) using sterile loops, and after growing in an incubator for 24 h under CO_2_, the pure cultures were obtained using the streaking method. The bacteria were identified using Bruker MALDI Biotyper^®^ software on the ultrafleXtreme MALDI-TOF/TOF mass spectrometer (Bruker Daltonics, Bremen, Germany) equipped with a modified Nd:YAG laser (Smartbeam IITM) operating at the wavelength of 355 nm and the frequency of 2 kHz and used to acquire spectra from strains of bacteria. Identified bacterial strains were deposited at − 80 °C using Microbank^®^, which is a unique cryovial system incorporating treated beads and a special cryopreservation solution.

After deposition, all three investigated bacteria were cultivated in headspace 20 mL vials with magnetic polytetrafluoroethylene/silicon screw caps. First of all, the vials were filled with autoclaved BHIA (4 mL) under a laminar chamber and stood in a diagonal position to obtain slants with a solid medium. Then, the slants were inoculated with a sterile loop holding one of the beads with selected bacteria. The vials were closed with screw caps, previously treated with UV light to pre-sterilize the surface. The vials with content were placed in an incubator for 24 h at 37 °C.

After incubation, the vial was placed in the heater with a set temperature of 37 °C for the extraction procedure. Later, lab-made fiber was inserted into the vial for the headspace extraction for 20 min. Next, fiber was immediately transferred to the GC injector for 5 min of desorption at 220 °C. Such procedure was repeated for the three types of bacteria and extraction of VOCs from the pure media^18^.

### Human subject research

Ethical approval for this study was received by Institutional Review Board of Maria Sklodowska-Curie National Institute of Oncology in Gliwice (agreement number code KB/430-78/22, date of approval 26 June 2022). The study was conducted according to the approved guidelines outlined in the Declaration of Helsinki. Informed consent was obtained from all participants prior to enrolment in the study.

## Results and discussion

Previously, we reported the synthesis of SPME fiber based on polypyrrole^13^. The fiber showed adequate reproducibility and values of the limit of detection in the range 0.59–283.33 ng mL^−1^ for standard solutions of VOCs. Yet, further progress can be made beyond this point. Conductive polymers, in particular polypyrrole, are flexible materials for modifications^10^.

There are two ways to introduce modifications into the structure of conductive polymers. Firstly, it is the incorporation of different counter ions. It has been shown that even the size of the incorporated ion may affect the morphology of the polymer coating and, subsequently, its selectivity^19^. The second way is the introduction of applicable functional groups or co-deposition of nanomaterials, MOFs, ionic liquids, or other monomers to the polymer^11^.

Metal–organic frameworks possess high porosity and surface area, which is favourable for the preparation of extraction coatings. Besides, the chemical structure of MOFs may enable additional interactions between analytes and coating, which can contribute to the specificity of the fiber towards some analytes/groups of analytes and thus decrease the limit of detection. Hence, we hypothesized that introducing MOFs as modifications to the structure of polypyrrole may decrease the limit of detection. To the best of our knowledge, pure PPy coatings have different porosity and morphology depending on synthesis parameters^19^. The surface area of PPy films can reach more than 220 m^2^/g^13^.

### Surface characterization of the PPy@ZIF-8 coating

#### SEM

**Figure 1:**
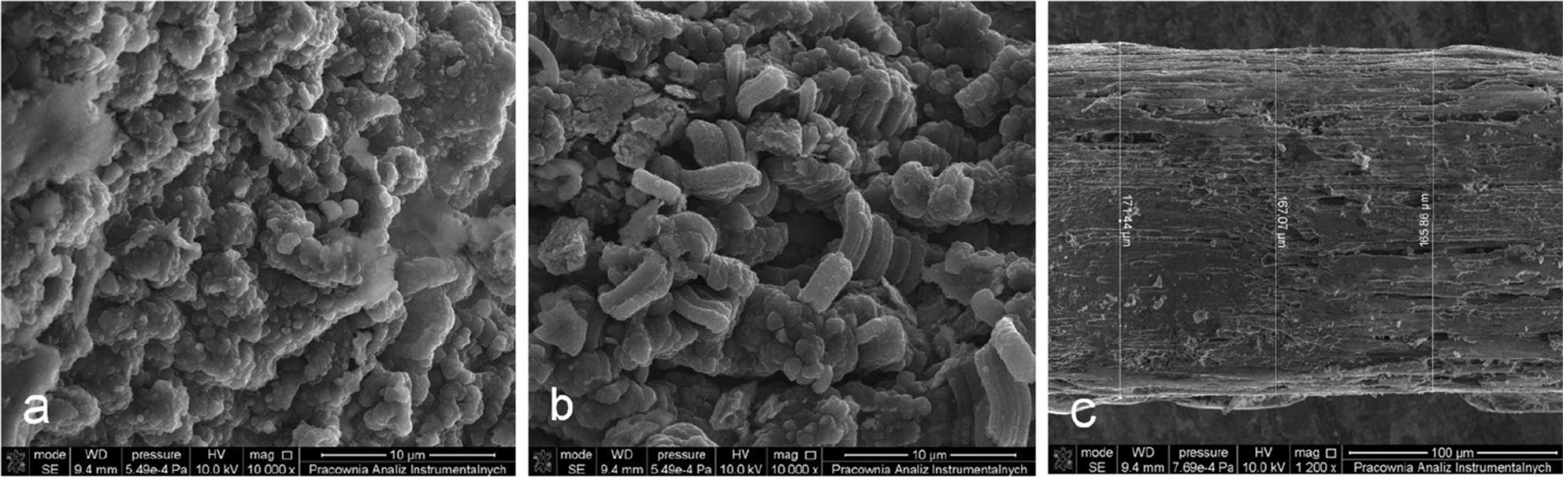
SEM images of the synthesized coatings: (**a**) PPy, (**b**) PPy@ZIF-8, (**c**) stainless steel support coated by PPy@ZIF-8.

SEM analysis showed the porous and nonhomogeneous structure of the obtained PPy@ZIF-8 film (Fig. 1b). Figure 1 demonstrated the differences in morphology of the coatings based on PPy and PPy@ZIF-8. Therefore, the incorporation of ZIF-8 into the polymer probably affected its structure (Fig. 1).

Since the film thickness of the coating may affect the extraction efficiency, the value of film thickness was estimated based on the SEM pictures and the well-known diameter of the stainless-steel substrate (Fig. 1c). The value of film thickness varied within the range between 65 and 72 µm.

#### FTIR-ATR spectroscopy

**Figure 2:**
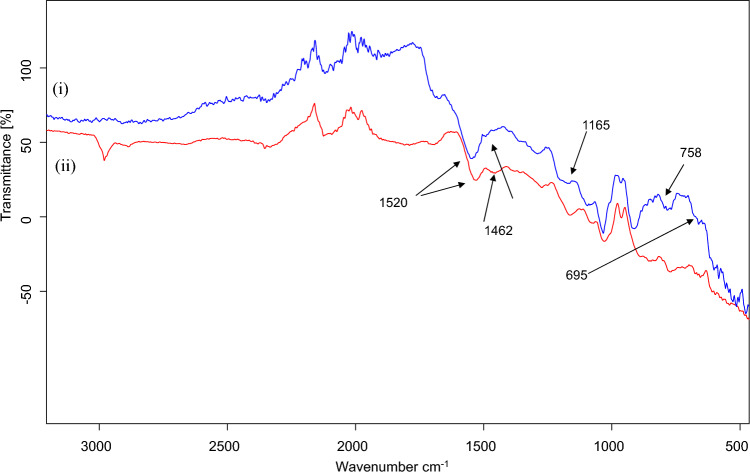
FTIR-ATR spectra of the synthesized coatings: (i) PPy@ZIF-8 and (ii) PPy.

The results of FTIR-ATR spectroscopy displayed the pyrrole ring band at ν = 1520 cm^−1^ for both PPy and PPy@ZIF-8 coatings (Fig. 4). Vibrations at ν = 1455 cm^−1^ for coatings (i) and (ii) may correspond to the formation of sulphonamides since vibrations in the range 1420–1370 cm^−1^ are characteristic of mono-n-substituted sulphonamide bond (–SO_2_–NH–)^20^. The spectra of PPy@ZIF-8 coating showed distinct bands at 1165, 695 and 758 cm^−1^ (Fig. 2). The band at 1165 cm^−1^ corresponds to the C–N bonds, the band at 758 cm^−1^ corresponds to the Zn–O bonds, and 695 cm^−1^ corresponds to Zn–N bonds in the imidazole group^21^. Hence, the spectra suggest the incorporation of ZIF-8 into PPy polymer.

### The study of the effect of parameters on the analytes’ responses

Crucial parameters that affect the electrochemical synthesis of nanocomposite based on polypyrrole and MOF are time, amount of MOF, and voltage. Variation of the voltage resulted in a loss of mechanical stability, which was observed by the loss of the coating in an attempt to insert it into the GC inlet. Hence, the effect of time and amount of MOF was studied at a constant value of voltage.

**Figure 3:**
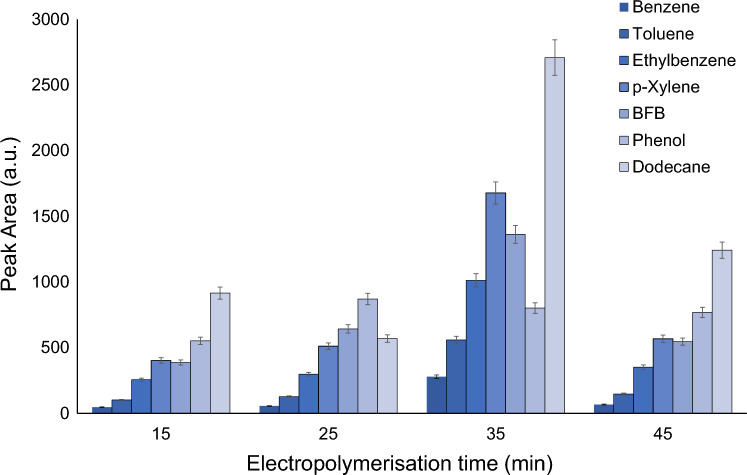
The effect of time of synthesis on responses of analytes extracted by coating based on PPy@ZIF-8.

According to obtained results (Fig. 3), the time of the synthesis affected the extraction performance of the coating based on polypyrrole and ZIF-8. The increase in time of synthesis improved the extraction efficiency for all analytes except phenol. The highest value of extraction performance was achieved at a time 35 min. A decreasing trend in signal intensity was observed for all analytes at a time 45 min. The highest response for phenol was obtained at a time 35 min (Fig. 3). In addition, even the synthesis time 15 min provided higher response as compared to previously reported PPy fiber^13^.

**Figure 4:**
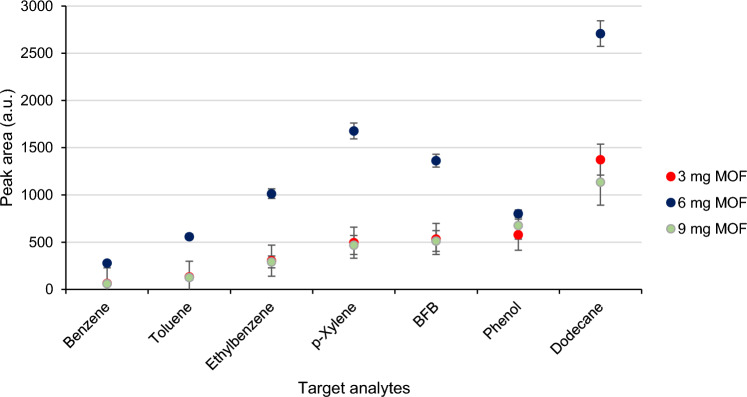
The effect of the amount of ZIF-8 added to the reaction mixture on responses of analytes extracted by coating based on PPy@ZIF-8.

The following parameter that affected the extraction performance is the amount of ZIF-8 that was added to the electrolyte-monomer solution. According to obtained results (Fig. 4), variation in the amount of added MOF provided significant differences in the extraction efficiency of resulting coating for some of the analytes. Figure 2 showed that 6 mg provided the highest responses for all analytes. The lowest values of RSDs were also obtained in case of addition of 6 mg of ZIF-8 to the reaction solution (Fig. 4).

The mass of ZIF-8 > 9 mg added to the reaction solution resulted in poor adhesion of the coating towards the stainless-steel wire. The mass < 3 mg resulted in the analytes responses similar to pure PPy coating, which probably indicated that ZIF-8 did not participate in composite formation.

Hence, the following parameters were selected for synthesis of PPy@ZIF-8: time 35 min, 6 mg of ZIF-8 and applied voltage 5 V.

### Utilization of obtained fiber for extraction of VOCs from standard solution

To the best of our knowledge, it is challenging to measure the mechanical stability of the fiber, and in particular, the adhesion between coating and support. For this reason, the responses of analytes and the corresponding value of standard deviation were considered for the assessment of fiber stability.

The values of the limit of detection (Table 1) and intra- and inter-assay reproducibility (Table 2) were calculated for PPy@ZIF-8 and PPy coatings. In order to assess the effect of PPy modification on LOD value, extraction of selected VOCs has been carried out at the same conditions that were optimized previously for PPy coating.

**Table 1:** The values of LOD for PPY and PPY@ZIF-8 coatings.

Analyte	PPy	PPy@ZIF-8	logk_ow_
	LOD, ng mL^−1^		[ref.]
Benzene	102.55	12.73	2.13^22^
Toluene	107.4	18.47	2.54^22^
Ethylbenzene	96.7	1.67	90.5^22^
*p*-Xylene	52.2	4.99	45.0^22^
BFB	0.71	0.09	0.59^23^
Phenol	298.68	18.14	8.0^24^
Dodecane	7.55	0.73	0.59^25^

**Table 2:** Intra- and inter-assay reproducibility for selected VOCs extracted using PPy@ZIF-8 coating.

Analyte	PPy@ZIF-8		PPy	
	Intra-assay	Inter-assay	Intra-assay	Inter-assay
	RSD, %		RSD, %	
Benzene	3.71	4.10	2.1	5.11
Toluene	3.95	2.66	1.1	6.58
Ethylbenzene	4.50	5.70	3.08	4.12
*p*-Xylene	4.43	5.82	5.74	6.08
BFB	6.94	11.65	4.22	3.18
Phenol	9.26	11.69	4.54	10.85
Dodecane	11.83	9.47	11.54	12.87

Table 1 showed significant differences in the values of LOD for extracted VOCs with the utilization of pure PPy and PPy@ZIF-8 coatings. The lowest value of the limit of detection was found for dodecane (Table 1). Such differences probably can be caused by the higher volatility of dodecane and the smallest value of its log k_ow_. In addition, Chang et al.^26^ suggested that the selectivity of ZIF-8 coatings for the *n*-alkanes probably can be explained by the suitability of the pores’ aperture.

Notably, the LOD values of selected VOCs for PPy@ZIF-8 coatings were found to be lower than those obtained for PPy coating, even at extraction conditions optimized for pure PPy coating. This means that the LOD value of PPy@ZIF-8 coating potentially can be found to be even lower than the values presented in Table 1.

The values of LOD for benzene homologs such as toluene, *p*-xylene extracted by PPy@ZIF-8 were found to be lower than for pure PPy coatings (Table 1). This probably can be explained by the general affinity of polypyrrole to benzene homologs due to π–π interactions^10^ and π–π stacking owing to the imidazole group of ZIF-8^26^.

Table 2 demonstrated that the values of relative standard deviation were found to be less than 12%. The data in Tables 1 and 2 indicated that modification of the PPy coating with ZIF-8 resulted in an increase in sensitivity for different groups of VOCs. The RSD value of the fiber-to-fiber reproducibility (inter-batch studies) is less than 15% for each chosen analyte. Reduction in extraction efficiency of PPy@ZIF-8 fibers was observed to the values in the range of 15–20% after 30 cycles (sorption/desorption) depending on analyte.

**Figure 5:**
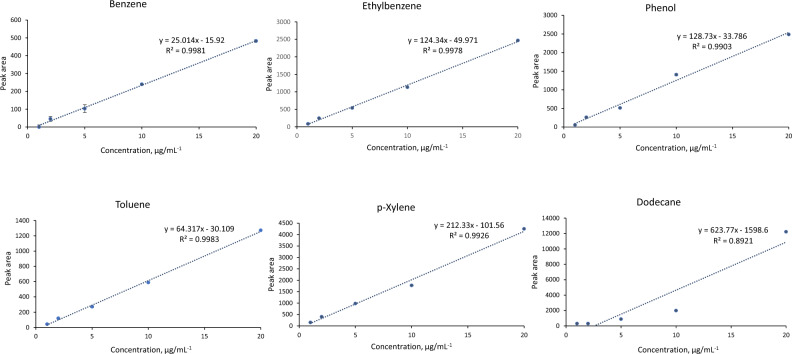
Calibration curves for 6 studied analytes extracted with utilization of PPy@ZIF-8 coating at the following parameters: 30 C, magnetic stirring at 750 rpm, 1 g of NaCl, pre-incubation time 17 min, extraction time 49 min.

The linear range has also been investigated within PPy@ZIF-8 fibers (Fig. 5). The linear range for all analytes accounted for 1–20 µg mL^−1^, except for dodecane. It can be explained by higher volatility of dodecane as compared to the rest of analytes as can be demonstrated by the value of logk_ow_ (Table 1).

### Extraction of VOCs emitted by bacteria with the utilization of the PPy@ZIF-8 coating

Literature data indicated that comparison of profiles of VOCs liberated by bacteria might assist in the identification and differentiation between the species^27,28^. In addition, monitoring of VOC profiles liberated by bacteria was reported as an alternative method to assess changes occurring in bacterial metabolism^29^. Therefore, we aimed to investigate the extraction performance of the PPy@ZIF-8 coating for VOCs liberated by three bacterial species.

Before starting the sample analysis, pre-conditioned fibers were exposed to the headspace of empty sterile vials, and blank analyses were carried out. In addition, culture media used for bacteria inoculation was also analysed. All signals originating from blank analyses (potentially fiber material) and culture media were excluded from identification and were not considered.

However, the effect of extraction time on VOCs’ responses emitted by bacteria was not studied in the current study since standard conditions used (extraction time 20 min) in case of PPy@ZIF-8 fibers provided reliable extraction performance. Since such parameters have a significant effect on extraction performance, the results obtained at standard extraction conditions potentially indicated that study of the effect of parameters on extraction performance for selected bacterial species may show improvement in extraction performance for those species, which emit VOCs at low concentration levels.

A total number of 405 volatile organic compounds were observed for three bacterial species and culture media. After the removal of signals originating from blank analyses and culture media, a total of 140 compounds was observed for 3 species such as *Hafnia alvei, Proteus mirabilis* and *Enterococcus faecalis*. After separation, different groups of VOCs were identified, and the predominant groups of volatiles were: hydrocarbons, ketones, alcohols, short-chain fatty acids, and sulphur-containing compounds.

**Figure 6:**
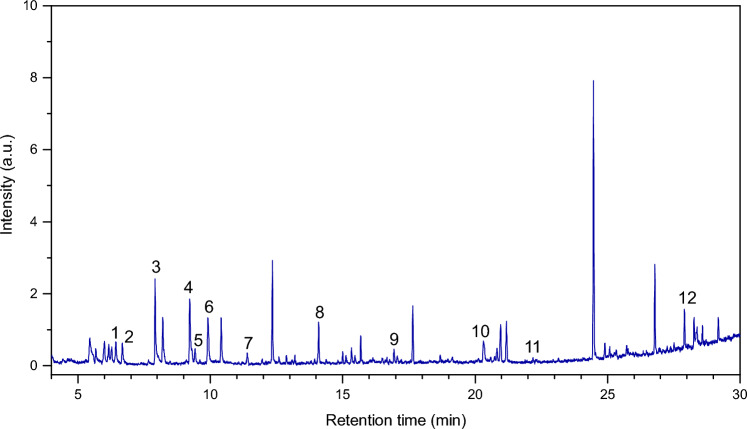
GC–MS chromatogram of VOCs liberated by *Enterococcus faecalis* extracted with the utilization of PPy@ZIF-8 coating.

Chromatogram corresponding to *Enterococcus faecalis* is presented in Fig. 6 as an example.

The list of identified compounds in the case of *Enterococcus faecalis* is shown in Table 3. The complete list of compounds including those originating from culture media and blank analyses is shown in Table S1. GC–MS chromatograms of VOCs liberated by *Hafnia alvei*, *Proteus mirabilis* and *Morganella morgani* can be found in Supplementary Information (Figs. S1–S3).

**Table 3:** Volatile organic compounds liberated by *Enterococcus faecalis.*

Peak	Compound	RT, min	Area
1	Ethylbenzene	6.425	106,199.3
2	*p*-Xylene	6.684	101,417.6
3	Cyclobutanol	7.666	21,150.53
4	Benzaldehyde	9.225	367,384.2
5	Dimethyl trisulfide	9.425	69,062.53
6	Phenol	9.907	273,315.6
7	Cathinone	11.966	19,642.11
8	1-Octanamine, N-methyl-	14.378	19,088.77
9	d-Alanine	16.489	17,278.31
10	2,4-Di-tert-butylphenol	21.183	165,678.2
11	Ethyne, fluoro-	23.148	10,694.75
12	Heptacosane	27.906	126,643.2

To the best of our knowledge, it is challenging to determine specific VOCs for bacterial species for several reasons. However, despite differences in culture media and methods, VOCs identified in this study partially agree with previously conducted research.

Nowadays is no recognized specific volatile compound dedicated to *Enterococcus faecalis.* However, several research groups proposed sets of VOCs, which were distinct and hence, potentially may serve as biomarkers for *Enterococcus faecalis* differentiation*.* Storer and co-authors measured VOCs liberated by bacteria in urine samples by SIFT-MS. Such VOCs as 2-pentanone, acetone, 2-methylbutanal, ammonia, dimethyl disulphide, dimethyl sulphide, ethyl butanoate, formaldehyde, hydrogen suphide, methyl mercaptan and propyl acetate were proposed as potential biomarkers^27^.

Another study has described the application of IMR-MS coupled with a headspace autosampler as a system to identify VOC liberated by *Enterococcus faecalis.* They carried out the measurements after incubation for 4, 8, and 24 h. Propanal, propanol, acetone, ethanol, isoprene and 1,3-butadiene were detected^28^.

## Conclusion

In this study, we presented a facile, cost- and labour-effective method for the synthesis of SPME fiber based on polypyrrole@ZIF-8 composite. The effect of the time of synthesis and mass of ZIF-8 on the sensitivity and stability of the coating was investigated. Direct electropolymerization of the PPy@ZIF-8 coating onto stainless steel wire allowed for the synthesis of the composite in one step. Such an approach prevented time-consuming and laborious synthesis protocols described in the literature.

Physical–chemical characterization of PPy@ZIF-8 coating has been carried out with utilization of scanning electron microscopy and FTIR-ATR spectroscopy. The values of limit of detection and intra- and inter-day reproducibility were found to be lower as compared to pure PPy coating for different groups of analytes, suggesting the validity of the main hypothesis of the current investigation. Furthermore, a significant increase in the sensitivity of PPy@ZIF-8 coating allowed for the profiling of VOCs liberated by bacteria.

Hence, PPy@ZIF-8 showed potential to be applied as a tool for the study of bacteria VOCs profiles.

## Supplementary Information

Supplementary Information.

## Data Availability

The datasets used and/or analysed during the current study available from the corresponding author on reasonable request.
