# Supplementary material for: Electropolymerized polypyrrole-MOF composite as a coating material for SPME fiber for extraction VOCs liberated by bacteria
Source: Sci Rep. 2023 Jun 1;13:8933. doi: 10.1038/s41598-023-36081-9 (PMC10235416; doi:10.1038/s41598-023-36081-9)
Supplement: Supplementary file 1 — Supplementary Information. [file 41598_2023_36081_MOESM1_ESM.pdf]

Electronic supplementary information

Electropolymerized polypyrrole-MOF composite as a coating material for SPME fiber for extraction VOCs liberated by bacteria

Radik Mametov [a,*], Gulyaim Sagandykova [a], Maciej Monedeiro-Milanowski [a], Dorota Gabryś [b] and Paweł Pomastowski [a]

[a] Centre for Modern Interdisciplinary Technologies, Nicolaus Copernicus University in Toruń, Wileńska 4, 87-100 Toruń, Poland

[b] Radiotherapy Department, Maria Sklodowska-Curie National Research and Institute of Oncology, Gliwice, Poland

*****corresponding author: mametov.radik@gmail.com; tel.: +48 56 665 60 01

Table of contents

Table S1. The complete list of volatile organic compounds liberated by *Enterococcus faecalis*

Figure S1. GC-MS chromatogram of VOCs liberated by *Hafnia alvei* extracted with the utilization of PPy@ZIF-8 coating

Figure S2. GC-MS chromatogram of VOCs liberated by *Proteus mirabilis* extracted with the utilization of PPy@ZIF-8 coating

Figure S3. GC-MS chromatogram of VOCs liberated by *Morganella morgani* extracted with the utilization of PPy@ZIF-8 coating

Table S1. The complete list of volatile organic compounds liberated by *Enterococcus faecalis*

#	Compound	*RT, min*	Area
1	Carbon dioxide	1.178	29161134
2	Acetic acid ethenyl ester	1.525	43362.78
3	Acetic acid	1.69	304000.4
4	unknown	2.066	2081327
5	1-Butanol	2.196	169935.3
6	4-Fluorohistamine	2.584	13022.96
7	Ethanol, 2-(vinyloxy)-	2.825	150875.8
8	Propanedioic acid	3.114	33640.5
9	Dextroamphetamine	3.831	30346
10	4-Fluorohistamine	3.908	26884.6
11	unknown	4.002	32725.73
12	unknown	4.443	33749.66
13	Propanedioic acid, propyl-	4.608	18519.68
14	2-Hexanamine, 4-methyl-	4.749	10720.93
15	Cyclotrisiloxane, hexamethyl-	5.449	245736.3
16	Cyclotrisiloxane, hexamethyl-	5.672	60856.85
17	Propanedioic acid, propyl-	6.007	98621.59
18	Propanedioic acid, propyl-	6.166	66512.75
19	Butanoic acid, 2-methyl-	6.278	62352.25
20	Ethylbenzene	6.425	106199.3
21	p-Xylene	6.684	101417.6
22	Cyclobutanol	7.666	21150.53
23	Oxime-, methoxy-phenyl-	7.913	374213.8
24	p-Bromofluorobenzene	8.207	211543.2
25	unknown	8.472	4799.06
26	unknown	9.101	12439.67
27	Benzaldehyde	9.225	367384.2
28	Dimethyl trisulfide	9.425	69062.53
29	Norpseudoephedrine	9.607	9738.16
30	Phenol	9.907	273315.6
31	Cyclotetrasiloxane, octamethyl-	10.407	202931.7
32	Amphetamine	11.395	50521.98
33	Cathinone	11.966	19642.11
34	Cyclotrisiloxane, hexamethyl-	12.342	338050.5
35	2-Hexanamine, 4-methyl-	12.589	20441.72
36	unknown	12.872	27296.15
37	unknown	13.095	14798.06
38	2,4-Dihydroxybenzaldehyde, 2TMS derivative	13.195	26162.09
39	Benzeneethanamine, N-methyl-	13.936	12360.14
40	Cyclopentasiloxane, decamethyl-	14.095	158207.7
41	1-Octanamine, N-methyl-	14.378	19088.77
42	2-Hexanamine, 4-methyl-	14.825	6835.65
43	Piperazine, 2-methyl-	15.001	35131.62
44	unknown	15.125	29181.77
45	unknown	15.336	56443.19

46	2-Pyrrolidinone, 4-hydroxy-5-methyl- (isomer 1)	15.466	25624.03
47	Cyclotetrasiloxane, octamethyl-	15.678	96311.66
48	unknown	16.136	23473.91
49	D-Alanine	16.489	17278.31
50	unknown	16.672	12023.46
51	R-(-)-Cyclohexylethylamine	16.777	13133.77
52	unknown	16.942	40786.19
53	1-Octanamine, N-methyl-	17.066	22490.58
54	2-Hexanamine, 4-methyl-	17.213	9221.67
55	Cyclohexasiloxane, dodecamethyl-	17.648	194395.5
56	unknown	18.689	24895.21
57	(+)-2-Aminoheptane	18.971	9842.74
58	Carbamic acid, monoammonium salt	19.148	21915.87
59	Imidazole, 2-amino-5-[(2-carboxy)vinyl]-	20.313	151372.8
60	R-(-)-Cyclohexylethylamine	20.595	14830.48
61	Phenethylamine, p,α-dimethyl-	20.748	20108
62	1-Octadecanamine, N-methyl-	20.83	54771.64
63	Cycloheptasiloxane, tetradecamethyl-	20.966	164018.6
64	2,4-Di-tert-butylphenol	21.183	165678.2
65	Cycloheptasiloxane, tetradecamethyl-	22.189	15622.62
66	Ethyne, fluoro-	23.148	10694.75
67	unknown	24.071	22670.02
68	Cyclooctasiloxane, hexadecamethyl-	24.471	951955.5
69	1-Octadecanamine, N-methyl-	24.901	51689.36
70	N-dl-Alanylglycine	25.083	24888.63
71	unknown	25.324	19467.15
72	unknown	25.718	30994.18
73	unknown	25.777	21091.79
74	unknown	26.048	11778.77
75	unknown	26.348	5859.37
76	Cyclononasiloxane, octadecamethyl-	26.789	298096.8
77	unknown	26.953	15714.04
78	unknown	26.989	8679.1
79	unknown	27.253	13150.25
80	unknown	27.383	23925.87
81	unknown	27.518	23147.88
82	Heptacosane	27.906	126643.2
83	unknown	28	5689
84	Benzeneethanamine, 2,5-difluoro-β,3,4-trihydroxy-N-methyl-	28.265	87941.69
85	Pterin-6-carboxylic acid	28.377	118690.3
86	Octasiloxane, 1,1,3,3,5,5,7,7,9,9,11,11,13,13,15,15-hexadecamethyl-	28.583	58191.11
87	unknown	28.718	11829.61
88	Cyclic octaatomic sulfur	29.183	78684.46
89	unknown	29.459	4502.86

90	unknown	30.124	39304.5
91	unknown	31.477	35453.79
92	unknown	34.535	388527.5

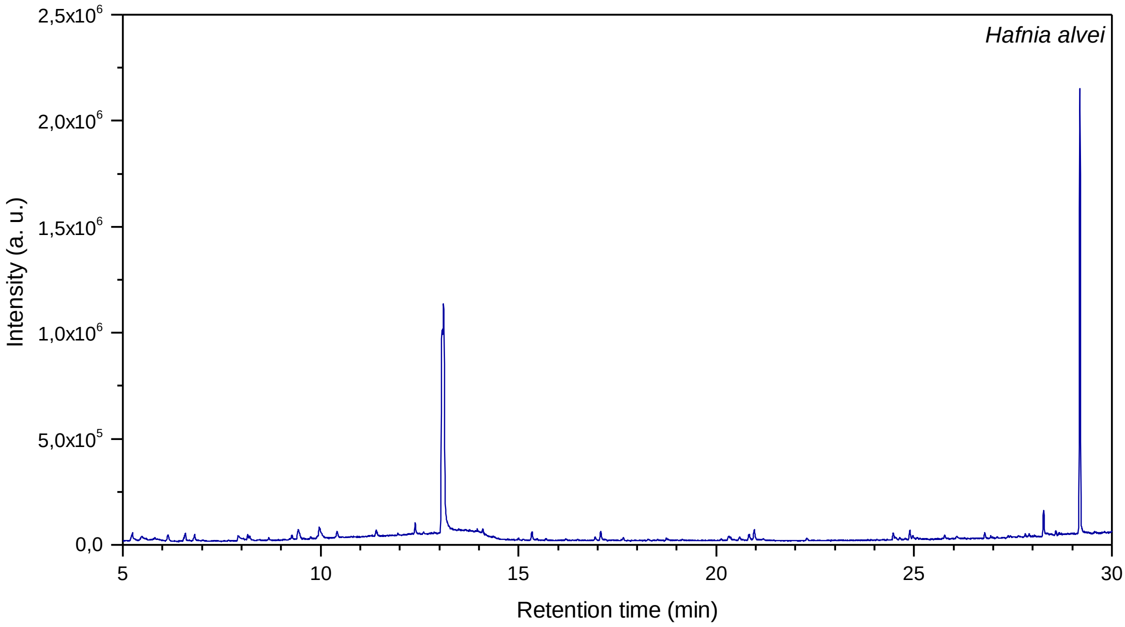

Figure S1. GC-MS chromatogram of VOCs liberated by *Hafnia alvei* extracted with the utilization of PPy@ZIF-8 coating

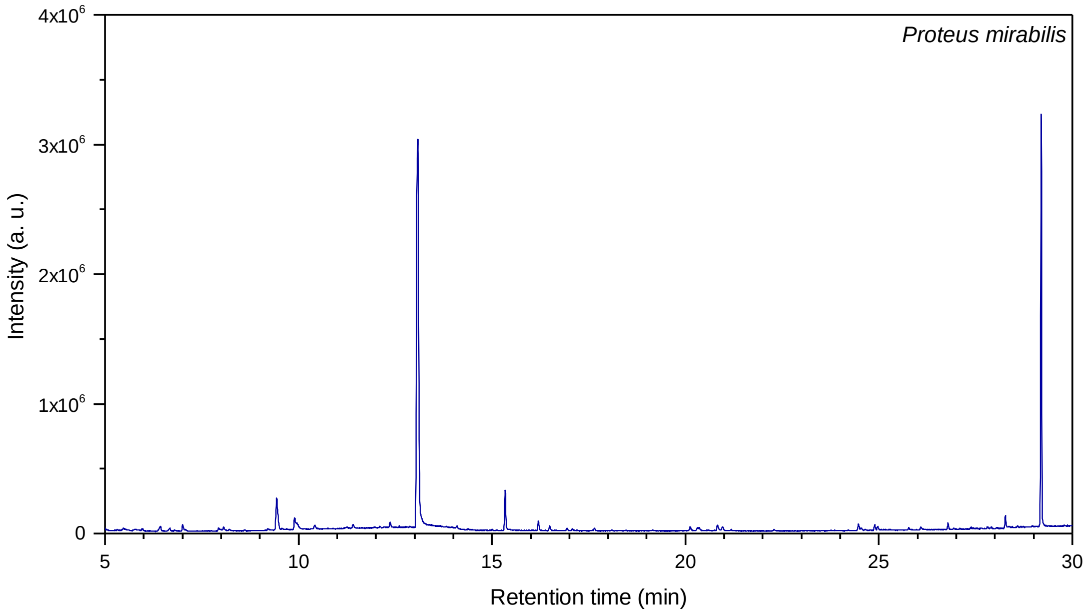

Figure S2. GC-MS chromatogram of VOCs liberated by *Proteus mirabilis* extracted with the utilization of PPy@ZIF-8 coating

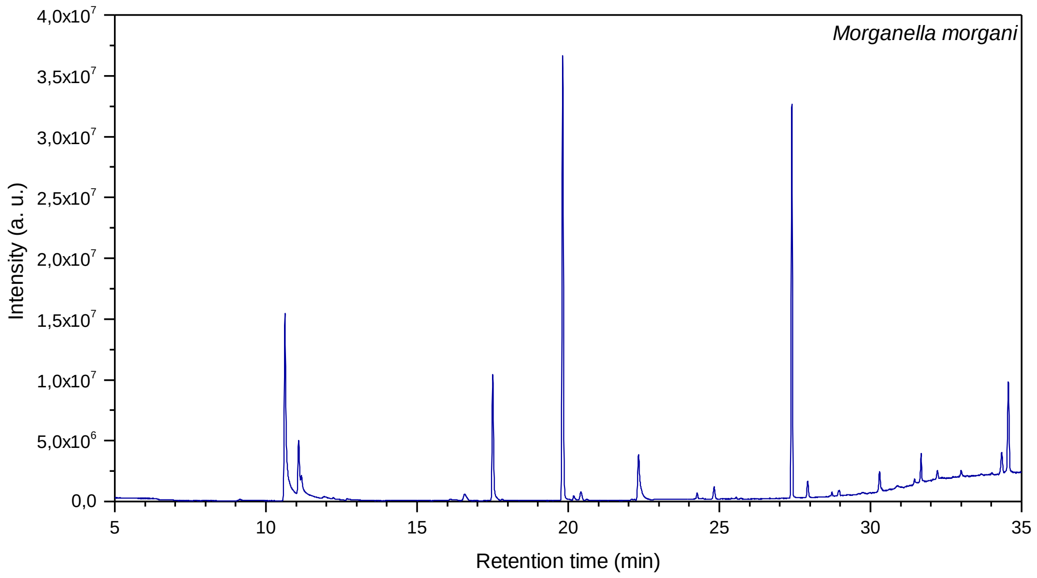

Figure S3. GC-MS chromatogram of VOCs liberated by *Morganella morgani* extracted with the utilization of PPy@ZIF-8 coating
